# Long-term functional outcomes in polytrauma: a fundamentally new approach is needed in prediction

**DOI:** 10.1007/s00068-023-02430-6

**Published:** 2024-02-15

**Authors:** Simone Meakes, Natalie Enninghorst, Natasha Weaver, Benjamin M. Hardy, Zsolt J. Balogh

**Affiliations:** 1 Department of Traumatology, John Hunter Hospital and University of Newcastle, Newcastle, NSW 2310 Australia; 2 Injury and Trauma Research Program, Hunter Medical Research Institute, Newcastle, Australia; 3 University of Newcastle, Newcastle, NSW Australia

**Keywords:** Polytrauma, SF36, Postinjury

## Abstract

**Purpose:**

Modern trauma care has reduced mortality but poor long-term outcomes with low follow-up rates are common with limited recommendations for improvements. The aim of this study was to describe the impact of severe injury on the health-related quality of life, specifically characterise the non-responder population and to identify modifiable predictors of poorer outcomes.

**Methods:**

Five-year (2012–2016) prospective cohort study was performed at a level 1 trauma centre. Baseline Short-Form Health Survey (SF36) was collected at admission, and at 6 and 12 months postinjury together with demographics, injury mechanism and severity, psychosocial wellbeing, and return to work capacity.

**Results:**

Of the 306 consecutive patients [age 52 ± 17 years, male 72%, ISS 21 (17, 29), mortality 5%], 195 (64%) completed questionnaires at baseline, and at 12 months. Preinjury physical health scores were above the general population (53.1 vs. 50.3, *p* < 0.001) and mental health component was consistent with the population norms (51.7 vs. 52.9, *p* = 0.065). One year following injury, both physical health (13.2, 95% CI 14.8, 11.6) and mental health scores (6.0, 95% CI 8.1, 3.8) were significantly below age- and sex-adjusted preinjury baselines. Non-responders had similar ISS but with a lower admission GCS, and were more likely to be younger, and without comorbidities, employment, or university education.

**Conclusion:**

Contrary to their better than population norm preinjury health status, polytrauma patients remain functionally impaired at least 1 year after injury. The identified high risk for non-responding group needs more focused efforts for follow-up. A fundamentally different approach is required in polytrauma research which identify modifiable predictors of poor long-term outcomes.

**Supplementary Information:**

The online version contains supplementary material available at 10.1007/s00068-023-02430-6.

## Background

Advances in prehospital care and robust trauma systems over the past decade have improved the likelihood of surviving serious injury [1]. However, improvements in hospital mortality have plateaued, with trauma survivors now experiencing unique challenges in returning to their preinjury health [2–11].

Traumatic injuries are the leading cause of long-term morbidity in the severely injured [3, 4]. Injury related mortality has declined but the long-term effects of surviving serious injury such as functional impairment and disability, persistent pain, and the psychological sequelae of trauma continue for many years after the initial injury [2, 4, 6, 12]. Post injury reduced physical and psychological capacity is closely linked with an increased likelihood of engaging in highrisk behaviours such as tobacco, drug use, excessive alcohol, poor nutrition, physical inactivity, sustained immune suppression and /or inflammation. These behaviours contribute to the burden of injury through the development of chronic diseases and worsening health related quality of life outcomes [13–17]. Injury burden measures must capture the long-term impacts of injury and identify the modifiable predictors for all survivors, including non- responders. Low response rates make it challenging to know how non-responders differ from responders in terms of recovery patterns as it unknown whether these patients have a poor, good, or equal health status compared to patients who participate in the study.

The primary aim of this prospective cohort study was to examine the health-related quality of life of severely injured trauma patients. Furthermore, we aim to establish the potentially modifiable independent predictors poor outcomes and to identify the specifics of the nonresponding population.

## Materials and methods

### Study design

The study was a prospective cohort study of adult trauma patients admitted to a level 1 major trauma centre from January 2012 to December 2016.

### Study setting

Located outside of Sydney metropolitan area, this level 1 major trauma centre geographically covers an area the size of England, estimated at 131,785 km^2^ with a resident population of approximately 920,370 people. It is the busiest trauma centre in NSW and services a unique district that encompasses a major metropolitan centre, regional communities, and a smaller percentage of people located in remote communities. The trauma centre treats over 630 severely injured adult patients per year with a mean age of 52 years and a median ISS of 21 and has progressively achieved hospital and 30-day mortality rate of 8%, keeping in line with state and national peers [2]. Blunt trauma accounts for 93% of the admissions. The most common mechanism of injury are falls (36%) followed by road trauma (33%) and all other injuries (11%) which include animal and agricultural related injuries.

### Ethical consideration

The study was approved by the hospital’s human research ethics committee (approval 08/02/20/5.02).

### Participant enrolment

All patients admitted were identified during routine daily rounds by Clinical Nurse Consultants. This cohort was a conveniency sample based on consecutive patient recruitment during office hours. Inclusion criteria included trauma patients aged 16 years or older, with Injury Severity Score (ISS) > 15, with an expected length of stay greater than 24 h. Exclusion criteria included patients less than 16 years old, superficial injuries that do not require specific management, dementia or significant pre-existing cognitive impairment or sustained severe traumatic brain injury affecting the ability to consent, or admitted with self-harm-related injuries.

During admission, patients completed a preinjury baseline health questionnaire and demographic and injury characteristics questionnaires. Patients’ long-term outcomes were assessed at 6 and 12 months postinjury by mail. If two attempts to make contact failed, two follow-up phone calls were made to complete the questionnaires over the phone. If the participant was unable to be contacted at 6 months, they were contacted at 12 months follow-up. Participants were considered lost to follow-up if they were uncontactable or declined to participate. The final evaluation included all survivors of major trauma who completed the questionnaires at 12 months follow-up.

#### Socio-demographic study factors

Socio-demographic factors including age, gender, working capacity, highest education level, residential postcodes, the number of comorbidities, and compensable status were collected. Current work status was asked at each follow-up, with additional variables such as full/light duties and full-time or part-time work. The highest education level attained was categorised into university or post graduate level vs. secondary or TAFE/apprenticeship level. Residential postcodes at the time of injury were used to generate the Index of Relative Socioeconomic Disadvantage (IRSD), which summarises the economic and social conditions within a particular area/postcode. Levels of disadvantage were collapsed into quintiles ranging from 1 (most disadvantaged) to 5 (least disadvantaged) to ensure a meaningful assessment of an association between IRSD and health outcomes [18]. Residential postcodes also mapped Accessibility/Remoteness index of Australia (ARIA), which classifies regions of Australia into five levels of remoteness (major cities, inner regional, outer regional, remote, and very remote). Co-morbid status of the participants was defined using the Charlson Comorbidity Index (CCI), mapped from the International Classification of Diseases 10th Revision Australian Modification (ICD_10_AM) diagnosis codes ranging from zero to six with a CCI of zero representing no CCI condition.

In NSW, compensation following motor vehicle accident was available under third party or ‘no fault’ principles, covered by the Motor Accident Authority (MAA), a government insurance regulator of Compensatory Third-Party Insurance (CTP) personal injury scheme [19]. Third party or a ‘no fault’ compensable status provides payments for medical treatment, rehabilitation expenses, loss of income (including past and future), and long-term support services which are paid as lump sums at claim settlement [19–22].

Death after discharge was collected using the district wide medical record and the NSW Birth, Death, and Marriage Registry.

#### Injury-related study factors

The Abbreviated Injury Scale (1–6, AIS) is an anatomically based injury severity scoring system that ranks injuries to a particular body region on a scale of one to six (6 is not survivable). The Injury Severity Score (1–75, ISS) was used as measures of injury severity and is calculated using the sum of the squares of the three highest AIS scores from three different body regions. Other injury-related study factors collected were mechanism of injury, polytrauma (AIS > 2 in more than two body regions), initial Glasgow Coma Score (GCS) on admission, intensive care unit length of stay (ICU LOS) and hospital length of stay (HLOS), and discharge disposition or death [23, 24].

### Health-related quality of life

#### SF36 version 2

HRQOL was assessed using the SF36 version 2, a validated tool for outcome research for trauma patients [25, 26]. SF36 is a generic tool consisting of 2 main scores, physical component score (PCS) and the mental health component score (MCS), which utilises 36 questions to summarise both the physical and mental health of the individual [27]. Within the physical and mental health components, there are four subdomain scores. Subdomains of PCS include physical functioning (PF) which scores the ability to perform physical tasks, role physical (RF) which scores physical limitations to daily activities, bodily pain (BP) which measures the extent in which pain interferes with work, and then overall general health (GH) [5, 27].

Subdomains of MCS include vitality (V) which measures perceived energy levels, social functioning (SF) which determines the extent physical and emotional health impacts social activities, role emotional (RE) which measures limitations in performance of daily activities due to emotional problems, and overall mental health (MH) [5, 27]. Each answer is given a pre-set value on a scale of 0 to 100 corresponding to a specific domain. Proprietary scoring software for SF36 version 2 (Quality Metric, Lincoln, USA) was used and meaningful comparisons regarding health status between trauma patients and Australian population norms were calculated [26, 27]. The Australian population norms calculated for the PCS and MCS were 50.27 and 52.92, respectively [26]. HRQOL was measured on admission to get an indication of the study participants’ preinjury health status, and then at 6 and 12 months follow-up after discharge from hospital.

#### Statistical analysis

Quantitative variables describing patients and their injuries were summarised as mean and standard deviation and/or median and inter-quartile range for skewed distributions. Categorical variables were summarised as frequency count and percentage for each level.

The scoring algorithm for SF36 was standardised for the Australian population using Stata syntax supplied [26] to calculate the two component scales and eight subdomain scores. Summary scores for standardised PCS and MCS were presented as mean and standard deviation [26]. Component scores were unable to be calculated for patients who were missing one or more items.

The main analysis was to estimate change in standardised component scale scores and subdomains from baseline to 6 months and from baseline to 12 months. Linear regression models were fitted within a generalized estimating equations (GEE) framework to estimate mean differences at each follow-up relative to baseline, using the robust variance estimator adjusted for repeated measures on patients. Sex and age at baseline were included as covariates to reduce confounding bias. Linear mixed models with a random intercept for patient were also fitted and the parameter estimates were similar. Model results were presented as mean differences with 95% confidence intervals and *p*-values from the GEE.

The second analysis was to determine predictors of component scale scores and whether any were associated with change over time (baseline to 6 months/12 months). Independent factors associated with long-term trauma outcomes such as age, sex, preinjury health status, ISS, hospital length of stay (HLOS), nature of injury, number of comorbidities, compensable status, and socioeconomic status have been described in the literature to be associated with HRQOL after injury [5, 8, 11, 28–31]. Univariate associations between potential predictors and component scale scores were modelled first, followed by a multivariable linear regression model that included all potential predictors. Interactions of potential predictors with time were also investigated.

Statistical significance was set at the 0.05 level. Data manipulation and statistical analyses were performed using Stata 14 and SAS 9.4 (SAS Institute, Cary, NC, USA) software. No data amputation methods were used for SF36 outcomes for missing data.

## Results

**Fig. 1:**
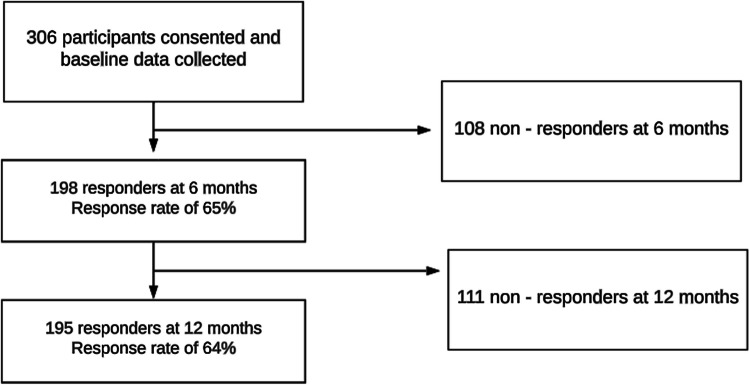
Patient selection. Flow chart showing follow-up patients and response rate at baseline, 6 months, and 12 months

Three hundred and six trauma patients were identified as eligible, 220 were males (72%) and 86 (28%) were females; Fig. 1 shows 306 enrolled patients, of which 195 patients completed follow-up questionnaires at 12 months, giving an overall follow-up rate of 64%. Study participants were similar in characteristics to the total cohort except regarding age (*p* < 0.001), initial lower GCS on admission (*p* = 0.005), employment prior to injury (*p* < 0.001), lower education (*p* = 0.017), and number of comorbidities (*p* < 0.001). Table 1 compares the baseline characteristics of those participants who were lost to follow-up (non-responders *n* = 111), and those participants who completed data collection at 12 months follow-up (responders *n* = 195). There were no significant differences between responders and non-responders with respect to gender, mechanism of injury, ISS, AIS, ICU and LOS, and hospital LOS. However, responders were significantly older (median ages 35 vs. 52 years), higher number of comorbidities, but less likely to have a low initial GCS on admission and more likely to be retired compared to the non-responders.

**Table 1:** Participants clinical and demographic characteristics

Patient characteristic	Responders (*n* = 195)	Non-responders (*n* = 111)	*p*-value
Age (years), median (IQR)	52 (37, 64)	35 (25, 47)	< 0.001
Age category, *n* (%)			< 0.001
Under 18 *n* (%)	1 (0.5%)	4 (2.8%)	
18–29	39 (20%)	39 (36%)	
30–44	31 (16%)	36 (32%)	
45–59	52 (27%)	21 (20%)	
60–74	64 (33%)	7 (6%)	
75 +	10 (5%)	4 (4%)	
Sex—male, *n* (%)	144 (74%)	76 (68%)	0.2623
Injury Severity Score (ISS), median (IQR)	21 (17, 29)	22 (17, 29)	0.970
Injury group AIS > 2, *n* (%)			
Head/neck	99 (51%)	68 (61%)	0.092
Chest	122 (63%)	73 (65%)	0.647
Abdomen/pelvis	99 (51%)	47 (42%)	0.137
Extremities	158 (81%)	92 (82%)	0.809
Polytrauma AIS > 2 in more than two body regions, *n* (%)	169 (86%)	95(85%)	0.654
Initial GCS in ED, *n* (%)			0.005
Mild or no TBI (GCS 13–15)	165(85%)	78 (70%)	
Moderate TBI (GCS 9–12)	10(5%)	8 (7%)	
Severe TBI (GCS 3–8)	20 (10%)	26(23%)	
Head Injury severity, *n* (%)			0.647
Head AIS 1–2	60 (31%)	43 (38%)	
Head AIS > 3	42 (22%)	26 (23%)	
Mechanism of injury, *n* (%)			0.558
Fall	38 (19%)	16 (14%)	
Cyclist	3 (1.9%)	1 (8%)	
Motorbike accident (MBA)	48 (25%)	34 (30%)	
Motor vehicle accident (MVA)	71 (36%)	45 (40%)	
Pedestrian	16 (8%)	5 (4%)	
Other	18 (11%)	10 (10%)	
Insurance status, *n* (%)			0.152
Compensable	69 (33%)	30 (27%)	
Non-compensable	130 (67%)	81 (73%)	
Medicare	66 (51%)	65 (79%)	
Private	64 (49%)	17 (21%)	
Hospital LOS, median (IQR)	20 (10, 39)	17 (10, 36)	0.234
ICU LOS, median (IQR)	0 (0, 4)	2 (0, 4)	0.475
Charlson Comorbidity Index, median (IQR)	1 (0, 3)	0 (0, 1)	< 0.001
None	82 (42%)	83 (74%)	
1	31 (16%)	13(12%)	
2 +	81 (42%)	15 (14%)	
Working prior to injury, *n* (%)			< 0.001
Working	118 (60%)	70 (63%)	
Unemployed	8 (4%)	18 (16%)	
Retired/home	60 (31%)	12 (11%)	
Student/other	8 (4%)	11(9%)	
Education level, *n* (%)			0.017
< Year 10	72 (37%)	42 (38%)	
Year 11–12	36 (18%)	32 (29%)	
TAFE/apprenticeship	56 (29%)	30 (27%)	
University/post graduate studies	33 (17%)	7 (6%)	
Discharge disposition, *n* (%)			0.618
Home	111 (57%)	73 (64%)	
Rehabilitation	65 (33%)	26 (23%)	
Acute care	12 (6%)	4 (4%)	
District hospital	7 (4%)	8 (7%)	
Mortality—died, *n* (%)	14 (7%)	6 (5%)	0.245
Baseline SF-36			
Mental component score (MCS), mean (SD)	52.4 (10.9)	50.9 (11.3)	0.242
Physical component score (PCS), mean (SD)	52.3 (9.4)	54.1 (8.1)	0.077

Continuous data are presented as medians with inter-quartile range (IQR) or mean with standard deviation (SD). P-values for continuous variables are from Wilcoxon-Mann–Whitney tests or t-tests. Categorical data are presented as frequency counts and percentages. *p*-values for categorical variables are from chi-squared tests

For the 195 participants studied, the mean (SD) age was 52 (17), approximately equal proportions in each of the age groups: 17–30 years, 31–45 years, 46–60 years, and 61–73 years, with 10 patients aged greater than 74. The median ISS was 21 and the most common mechanism of injury was road traffic-related motor vehicle collision 36% (MVC) and motor bike collision 25% (MBC), followed by falls (19%), and pedestrians hit by vehicles (8%). Length of stay in ICU ranged from 0 to 37 days with mean 5.6 days and median 3 days. Total hospital length of stay ranged from 1 to 143 days with mean 27.2 days and median 20 days. Discharge disposition for most patients was home with specialist follow-up appointments (57%), rehabilitation facilities (33%), or transferred to acute care (12%) or district hospital (7%).

Almost all patients (98%) in this cohort were of English language background and over half of the sample had no post-secondary educational qualification with only 17% having university qualification and 29% with vocational training as their highest educational qualification. Before the injury, over 60% of the study participants were employed full-time/part-time, with unemployment rates being higher in the non-responder group compared to the responders’ group (16% vs. 4%). Thirty-one percent of responders were retired compared to only 11% in the non-responders group. Return to work (RTW) rates were 38% and 42% at 6 and 12 months, respectively, with only 39% returning to normal duties at 12 months and 33% working reduced hours. The proportion of patients who indicated full recovery increased incrementally from 6 to 12 months postinjury from 36 to 45%. The most common factors impeding recovery were pain 62%, loss of function 71%, and inconvenience 62%.

Health status outcomes using the SF36 v2 questionnaire were measured on admission, and then at the 6 and 12 months follow-up, scores standardised to the Australian general population are summarised in Table 2. Preinjury baseline health scores measured on admission reported mean values for Physical Component Scale (PCS) and Mental Component Scale (MCS) scores of 53.1 and 51.7, respectively (Table 2). The preinjury baseline PCS scores in this cohort were significantly higher than the Australian general population reporting scores of 53.1 compared to 50.3, with mean difference of 2.85 (95% CI 1.86, 3.84; *p* < 0.001). For PCS, the subdomain scores of physical functioning (*p* = 0.024), role physical (*p* = 0.011), bodily pain (*p* < 0.001), and general health (*p* < 0.001) were significantly higher preinjury compared to the Australian general population. However, preinjury baseline MCS scores compared to Australian general population were slightly lower, with preinjury baseline MCS scores of 51.7 compared to 52.9, with mean difference of –1.18 (95% CI –2.42, 0.08; *p* = 0.065). For MCS, the subdomain scores of vitality (*p* < 0.001) and role emotional (*p* = 0.047) were significantly higher preinjury compared to the Australian general population.

**Table 2:** Change in physical and mental functioning outcomes over time

Outcome	Australian norm* Mean (SD)	Baseline mean (SD)	6 months mean (SD)	12 months mean (SD)	Difference 6 mths—baseline Mean (95% CI)	*p*-value	Difference 12 mths—baseline Mean (95% CI)	*p*-value
Physical component score (PCS)	50.3 (9.7)	53.1 (8.8)	36.9 (10.7)	39.9 (11.4)	− 16.2 (− 17.8, − 4.6)	< .001	− 13.2 (− 14.8, − 11.6)	< .001
Proportion below AUS norm for PCS, *n* (%)		46 (29%)	136 (85%)	125 (78%)				
Mental component score (MCS)	52.9 (10.2)	51.7 (11.1)	44.3 (15.2)	45.8 (14.6)	− 7.5 (− 9.7, − 5.2)	< .001	− 6.0 (− 8.1, − 3.8)	< .001
Proportion below AUS norm for MCS, *n* (%)		57 (35%)	144 (89%)	134 (83%)				
Physical domains								
Physical functioning (PF)	50.6(9.2)	51.9 (10.0)	35.5 (12.7)	38.8 (12.6)	− 16.4 (− 18.3, − 14.5)	< .001	− 13.0 (− 14.9, − 11.2)	< .001
Role limitation–physical (RP)	50.7(9.8)	52.1 (9.3)	33.5 (12.8)	37.9 (12.8)	− 18.6 (− 20.5, − 16.7)	< .001	− 14.2 (− 16.0, − 12.4)	< .001
Bodily pain (BP)	52.2(9.0)	54.4 (10.1)	40.6 (10.8)	43.3 (11.2)	− 13.8 (− 15.4, − 12.1)	< .001	− 11.0, (− 12.8, − 9.3)	< .001
General health (GH)	50.5(10.4)	52.7 (8.6)	44.0 (10.7)	44.2 (11.2)	− 8.7 (− 10.2, − 7.2)	< .001	− 8.5 (− 10.1, − 7.0)	< .001
Mental domains								
Vitality (VT)	51.4(10.4)	55.3 (9.9)	44.8 (10.9)	45.4 (11.9)	− 10.6 (− 12.3, − 8.8)	< .001	− 9.9 (− 11.7, − 8.1)	< .001
Social functioning (SF)	50.8(9.4)	50.0 (10.4)	38.5 (13.2)	42.0 (12.9)	− 11.4 (− 13.4, − 9.5)	< .001	− 7.9 (− 9.8, − 6.0)	< .001
Role limitation–emotional (RE)	52.0(8.2)	50.8 (10.0)	39.2 (15.4)	41.5 (14.7)	− 11.6 (− 13.9, − 7.1)	< .001	− 9.3 (− 11.4, − 7.1)	< .001
Mental health (MH)	53.2(9.6)	52.7 (10.1)	45.1 (13.1)	46.6 (12.6)	− 7.6 (− 9.5, − 5.7)	< .001	− 6.1 (− 7.9, − 4.3)	< .001

Trends in SF36 v2 scores at baseline, 6 months, and 12 months. Models for change were estimated via linear regression in a GEE framework adjusted for repeated measures over three timepoints and included covariates for sex and age at baseline. *All models include random effect for patient estimated *via* REML*

At 6 months follow-up, participants showed significantly diminished mean PCS scores compared with the Australian general population. Mean PCS scores were reported as 36.9 with a mean score difference from baseline of 16.2 (95% CI 17.8, 14.6; *p* < 0.001). MCS at 6 months follow-up showed slightly improved results, with an overall mental health mean score of 44.3 with a mean score difference from baseline of 7.5 (95% CI 9.7, 5.2; *p* < 0.001). All mean scores of the 8 subdomains of SF36 were significantly lower compared to the Australian general population.

**Fig. 2:**
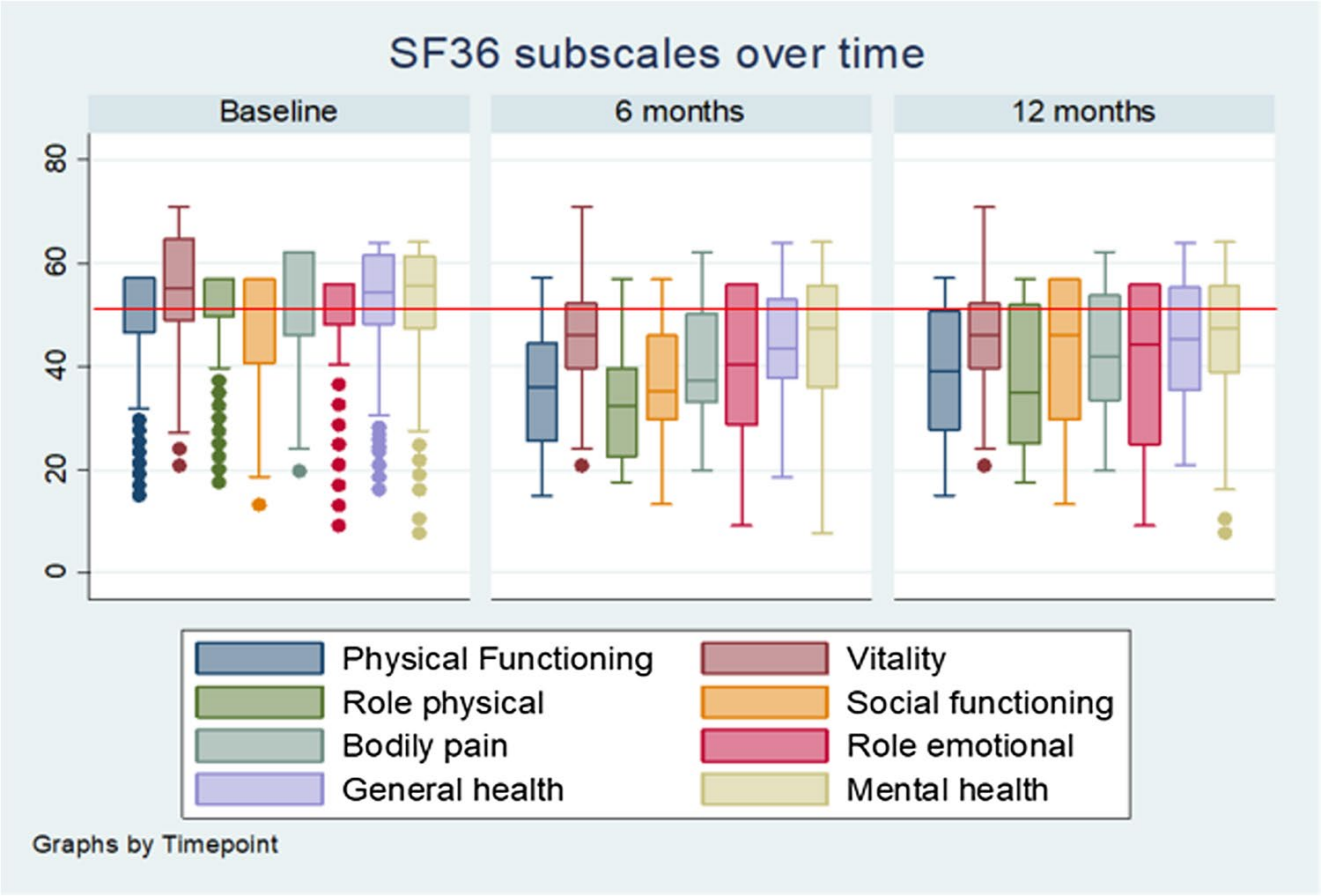
Distribution of domain scores of patients functioning over time. SF36 v2 subscales over time from baseline, 6 months, and 12 months. PCS physical component score, MCS mental component score. The red line indicates the Australian norms

Subsequently at 12 months follow-up, mean PCS scores remained significantly lower than preinjury baseline scores with a mean PCS of 39.9 and a mean difference from baseline of 13.2 (95% CI 14.8, 11.6; *p* < 0.001) showing only a slight improvement in scores from 6 months. MCS showed a slight difference in overall mental health from preinjury baseline with a mean MCS score of 45.8 and a mean difference from baseline of 6.0 (95% CI 8.1, 3.8; *p* < 0.001). MCS showed the lowest average decrease from baseline when compared to PCS at 12 months. Study participants continued to have significantly lower mean scores in all 8 subdomains with role physical (RF) having the largest average decrease at 12 months (Fig. 2) with a mean difference of 14.2 (95% CI 16.0, 12.4; *p* < 0.001), followed by physical functioning 13.0 (95% CI 14.9, 11.2; *p* < 0.001), bodily pain 11.0 (95% CI 12.8, 9.3; *p* < 0.001), and social functioning 7.9 (95% CI 9.8, 6.0; *p* < 0.001). Despite improvements in both HRQOL component scores, 78% and 82% of patients, respectively, had PCS and MCS mean scores well below the Australian general population at 12 months postinjury (Table 2).

Older age (*p* < 0.001), employment prior to injury (*p* = 0.006), number of comorbidities (*p* < 0.001), ISS (*p* = 0.010), polytrauma (*p* = 0.083), HLOS (*p* < 0.001), ICU admission (*p* = 0.022), and compensable status (*p* < 0.001) were significantly associated with lower physical functioning in the univariate analyses (Table 3). In the multivariable analysis, HLOS (*p* = 0.016), number of comorbidities (*p* = 0.005), and compensable status (*p* < 0.001) were associated with lower physical functioning. Similarly, for MCS, in the univariate analyses, HLOS (*p* < 0.001), ICU admission (*p* = 0.057), polytrauma (*p* = 0.057), low GCS on admission (*p* = 0.015), and compensable status (*p* = 0.119) were associated with lower MCS functioning. However, in the multivariate analysis, only HLOS (*p* < 0.0003) were associated with lower mental functioning.

**Table 3:** Variables associated with physical and mental functioning outcomes over time

Variable	Comparison	Unadjusted mean (95% CI)	Unadjusted *p*-value	Adjusted mean (95% CI)	Adjusted *P*-value	Interaction with time at 6 months Mean, *p*-value	Interaction with time at 12 months Mean, *p*-value
Outcome: physical component score (PCS)							
Age		− 0.16 (− 0.21, 0.11)	< .001	− 0.05 (− 0.14, 0.03)	0.199	0.01; *p* = 0.756	− 0.01; *p* = 0.860
Sex	Female vs. male	1.19 (− 0.92, 3.97)	0.268	1.01 (− 0.87, 2.90)	0.291	− 0.34; *p* = 0.831	− 2.07; *p* = 0.270
Education—university	Yes/No	1.08 (− 1.66, 3.81)	0.441	1.50 (− 0.92, 3.91)	0.224	1.53; *p* = 0.433	− 0.20; *p* = 0.931
Employment	Yes/No	2.65 (0.75, 4.55)	0.006	− 0.19 (− 2.14, 1.75)	0.847	− 2.31; *p* = 0.100	− 3.28; *p* = 0.051
Charlson Comorbidity Index (CCI)		− 1.86 (− 2.38, − 1.33)	< .001	− 1.35 (− 2.29, − 0.42)	0.005	0.33; *p* = 0.427	0.17; *p* = 0.719
Injury Severity Score (ISS)		− 0.12 (− 0.21, − 0.03)	0.010	− 0.04 (− 0.13, 0.06)	0.463	− 0.12; *p* = 0.071	− 0.24; ***p* = 0.002**
Hospital LOS		− 0.08 (− 0.11, − 0.04)	< .001	− 0.04 (− 0.08, − 0.01)	0.016	0.14; *p* < .001	− 0.12; ***p* < 0.001**
ICU admission	Yes/No	− 2.17 (− 4.04, − 0.31)	0.022	− 0.78 (− 2.72, 1.16)	0.428	− 3.40; *p* = 0.013	− 4.64; ***p* = 0.004**
Polytrauma	Yes/No	− 2.41 (− 5.14, 0.32)	0.083	− 1.95 (− 4.56, 0.65)	0.142	− 2.76; *p* = 0.188	− 5.62; *p* = 0.019
Initial GCS		0.11 (− 0.14, 0.37)	0.376	0.04 (− 0.22, 0.29)	0.782	0.20; *p* = 0.318	0.36; *p* = 0.144
Insurance—compensable	Yes/No	− 3.91 (− 5.87, − 1.96)	< .001	− 3.53 (− 5.34, − 1.72)	< .001	− 6.01; *p* = < .001	− 7.17; ***p* < .001**
Outcome: mental component score (MCS)							
Age		0.04 (− 0.03, 0.11)	0.304	0.08 (− 0.04, 0.20)	0.209	− 0.05; *p* = 0.338	− 0.01; *p* = 0.866
Sex—female	Female vs. male	1.51 (− 1.28, 4.31)	0.288	0.94 (− 1.85, 3.72)	0.510	3.04; *p* = 0.162	2.77; *p* = 0.202
Education—University	Yes/No	− 1.27 (− 4.90, 2.36)	0.493	− 1.52 (− 5.12, 2.08)	0.406	− 3.27; *p* = 0.220	− 1.00; *p* = 0.706
Employed	Yes/No	2.28 (− 0.27, 4.82)	0.079	2.67 (− 0.21, 5.54)	0.069	− 0.66; *p* = 0.732	0.47; *p* = 0.811
Charlson Comorbidity Index (CCI)		0.07 (− 0.69, 0.82)	0.866	− 0.17 (− 1.56, 1.22)	0.813	0.08; *p* = 0.888	− 0.01; *p* = 0.991
Injury Severity Score (ISS)		− 0.09 (− 0.21, 0.03)	0.134	0.01 (− 0.13, 0.15)	0.859	− 0.30; *p* = 0.001	− 0.11; *p* = 0.227
Hospital LOS		− 0.09 (− 0.13, − 0.04)	< .001	− 0.08 (− 0.13, − 0.03)	0.003	− 0.14; *p* < .001	− 0.14; ***p* < .001**
ICU admission	Yes/No	− 2.41 (− 4.90, 0.07)	0.057	− 0.05 (− 2.92, 2.83)	0.975	− 5.20; *p* = 0.006	− 4.32; *p* = 0.023
Polytrauma	Yes/No	− 3.51 (− 7.12, 0.10)	0.057	− 2.78 (− 6.62, 1.07)	0.157	− 2.63; *p* = 0.365	− 1.05; *p* = 0.706
Initial GCS		0.41 (0.08, 0.75)	0.015	0.26 (− 0.12, 0.64)	0.176	0.86; *p* = 0.001	1.04; *p* = 0.001
Insurance—compensable	Yes/No	− 2.12 (− 4.78, 0.54)	0.119	− 1.44 (− 4.13, 1.24)	0.292	− 9.11; *p* < .001	− 10.15; ***p* < .001**

Variables associated with physical and mental functioning outcomes over time. Effects were estimated using linear regression within a GEE framework adjusted for repeated measures over the three timepoints. Adjusted effects are from models that included all variables in the table. Models with interaction included an effect of the variable by categorical time

For the PCS outcome, there were significant negative interactions with time at 12 months compared to baseline for ISS (*p* = 0.002), hospital LOS (*p* < 0.001), ICU admission (*p* = 0.004), polytrauma (*p* = 0.019), and compensable status (*p* < 0.001) indicating that patients who were severely injured with more than two body regions injured, or had prolonged hospital LOS, with compensable status had larger decreases in physical functioning over time. For the MCS outcome, there was a significant negative interaction with time at 12 months compared to baseline for hospital LOS (*p* < 0.001), initial low GCS (*p* = 0.001), and compensable status (*p* < 0.001) indicating that patients with prolonged hospital LOS, a low initial GCS on admission, and compensable status had larger decreases in mental functioning over time. All other interactions with time for the two outcomes, particularly age and gender, were non-significant. The correlation between pre-injury PCS and MCS is very weak and non-linear (Pearson’s *r* = 0.10, Spearman’s rho =  − 0.03). At both follow-ups, the correlations are slightly higher but similar in value for Pearson’s and Spearman’s (*r* = 0.24 at 12 months).

## Discussion

The study of long-term recovery outcomes after traumatic injury has emerged as the benchmark for evaluating the quality of life among trauma survivors [4, 5]. Analyses of self-reported impairments in physical and emotional wellbeing attempt to capture and quantify the long-term burden of traumatic injury with the aim to improve quality trauma care and utilisation of health care resources. This study demonstrated significantly better preinjury physical health compared to the Australian general population. However, at 12 months follow-up, the HRQOL scores in both physical and mental health remained well below that of the Australian general population, with 78% and 82% of patients, respectively, had PCS and MCS mean scores well below the Australian general population. Socio-demographic and injury-related factors were found to influence patients’ recovery, with responder demographic characteristics describing an older population with increased comorbidities. After adjusting for age and other confounding variables, injury severity, polytrauma, low GCS on admission, ICU admission, hospital LOS, and compensable status were associated with persistent poor HRQOL at 12 months after injury in this cohort. Our responders revealed a high incidence of ongoing problems 12 months postinjury consistent with chronic disease status, which could be predicted only by non-modifiable factors by healthcare. This has significant implications for trauma centres, rehabilitation, and compensation schemes resulting in loss of productivity and increased health care utilisation. Injury burden measures need to capture the long-term impacts of injury and identify modifiable predictors for all survivors, including non-responders. Non-responders in this cohort were comparable to responders in regard to injury type and severity but were younger with considerably less comorbidities, had a lower education level, unemployed prior to injury, and admitted with a lower initial GCS. Similar to other studies, these low response rates make it challenging to know how non-responders differ from responders in terms of long-term health outcomes and recovery patterns. This study is the first to our knowledge to describe the 12-month functional outcomes after major trauma managed inclusively in our state, contributing to the growing evidence that demonstrates the long-term burden of traumatic injuries. Understanding disease burden and the complexity of factors impeding recovery adds insight into the feasibility of modifying interventions for the at-risk patients to reduced overall morbidity.

HRQOL outcomes in this study group reported preinjury scores above the general population with significant drops in both PCS and MCS scores below the general population at 12 months postinjury. Selection bias would account for preinjury scores above the general population as these patients were active enough to be severely injured. However, at 12 months postinjury, all HRQOL subdomains were impaired with role physical incurring the largest average decrease followed by physical functioning and bodily pain. Suggesting a substantial proportion of study participants reported persistent difficulties in performing regular physical activities due to poor physical health and bodily pain [27]. These trends in reduced physical functioning and role physical domains correlates with self-reported consequences of injury being persistent pain (62%), loss in function (71%), and incapable of returning to normal work 12 months after injury. Although only 45% of patients reported full recovery at 12 months, it appears that this slow recovery after injury is consistent with literature from national and international studies [3–5, 8–11, 28, 32, 33]. Soberg et al. reported outcomes on HRQOL outcomes on trauma patients over a 10-year period and found that physical functioning and bodily pain during the first years after injury were significant predictor of poor long-term physical health [34]. The independent predictors associated with reduced physical and mental health status scores found in this cohort appear to be relatively consistent with findings reported in national studies from Victoria, New South Wales, and Queensland [9, 11, 32]. Aitken et al. report outcome measures on 123 ICU trauma patients showing steady improvements in both MCS and PCS at 24 months, with longer hospital LOS as a predictor of lower physical functioning, with an average HLOS of 20.2 days and ICU LOS of 3 days [9]. Similarly, Gabbe et al.’s study of 662 Victorian major trauma patients monitored over 24 months showed improvements in PCS at 12 months postinjury, with MCS scores only improving after 18 months after injury [32]. In addition, Gabbe et al. identified poorer risk adjusted functional outcomes experienced by women, older patients with multiple comorbidities, those with lower education levels, and compensable cases [6, 33]. International studies also reported similar findings; Gunning et al. reported at 1-year follow-up PCS and MCS to be 45.6 and 47.2, respectively, for their 1870 Dutch trauma patients [5]. They found age, ISS, hospital LOS, ICU LOS, and severe injury to the head and extremities were associated with poorer outcomes at 12 months. Other specific patient factors or predictors associated with prolonged recovery in trauma include female gender, ICU admissions, injury type, and level of education [5, 6, 8, 10, 33, 35]. Haider et al. reported on 1736 US trauma patients with significantly diminished physical and mental health at 12 months compared to US general population [8]. Reporting independent factors associated with long-term outcomes as older age, females, lower education, number of comorbidities, and LOS significantly associated with worse recovery.

The implications of long-term physical and psychological burden of injury are currently being described through the literature as a precursor to the development of chronic physical health conditions. Chronic diseases are the leading cause of mortality, being responsible for 68% of all deaths worldwide [36]. The links between physical and psychological insults and chronic physical health conditions are multifaceted, with chronic health outcomes becoming more prevalent in those with severe injuries, reduced health-related quality of life, physical inactivity due to persistent pain and disability, unhealthy social habits, and high-risk behaviours, and/or chronic inflammation [13–17, 36, 37]. Although unable to describe the unhealthy social habits, in our participants at their 12 months follow-up, we found greater than 70% of participants had HRQOL well below the Australian general population, with polytrauma patients showing a larger decrease in physical functioning over time. This is consistent with the concept that polytrauma is a systemic disease (52) beyond the actual anatomical injuries described by the Injury Severity Score. Gelaw et al. reported 11% of patients with serious orthopaedic injuries experienced a significant burden of new onset chronic physical health conditions up to 5 years, with cancer, cardiovascular disease, and hypertension as the three most common new-onset conditions [13], while Stewart et al. found that the prevalence of hypertension, coronary artery disease, diabetes mellitus, and chronic kidney disease among injured combat service members in 14.3%,1.4%, 2.1%, 1.4%, respectively, during their follow-up period [15]. Age is also an important consideration with chronic physical health and older adults as the combined effect of severe injury, reduced HRQOL, and pre-existing comorbidities would increase the long-term burden of injury through multimorbidity, frequent health care utilisation, and complexity of management and recovery [13].

Of the 195 patients studied, just under a third of responders were over the age 60 years old, with 82% of those > 60 years having a Charlson Comorbidity Index (CCI) greater than 2. As the Australian population ages, the incidence of low falls increases and because of their frailty, multiple comorbidities, decreased physiological reserves, and stress owing to trauma and hospitalisation results in prolonged hospital LOS, ICU admission, and substantial morbidity [29, 38–40]. These factors highlight groups ‘at risk’ of poorer outcomes at 12 months which could potentially benefit from tailored interdisciplinary team approach in the management of complex needs impacting their recovery process after trauma.

Compensation for injury has frequently been reported to affect the trauma patient’s recovery and long-term outcomes [6, 20, 34, 41]. High levels of stress, anxiety, depression, reduced return to work rate, and longer recovery times have been associated with the use of compensation postinjury [31, 42, 43]. In this cohort, compensable status (*p* < 0.001) had a negative interaction with time at 12 months compared to baseline for both mean PCS (− 7.17; *p* < 0.001) and MCS (− 10.15; *p* < 0.001), with mental health being slightly worse. Primarily, a fault-based scheme, NSW CTP, is a complex process characterised by poor communication, delays to settlement claims, excessive paperwork, and disputes over liability causing extreme levels of stress [34, 41]. These findings add insights regarding the ongoing burden of injury and the need for better service provision planning for after acute care discharge to enable a quick return to optimal wellbeing.

The strength of this study is its prospectively longitudinal design and measurement of key demographic and injury-related data of both responder and non-responders. Comparable with other studies, non-responders were younger, with considerably less comorbidities, had a lower education level, were unemployed prior to injury, and admitted with a lower initial GCS [3, 5, 8, 12, 21, 29]. Gabbe et al. described lost to follow-up participants to be younger but were less severely injured and more likely to be injured in through self-harm or assault with a higher prevalence of mental health, drug, and alcohol conditions [13]. In this cohort, no statistical difference was noted in injury or hospital characteristics such as ISS, AIS, head injury severity, mechanism of injury, HLOS, and ICU LOS suggesting similarities between responders and non-responders regarding injury type, severity, mechanism of injury, and LOS. Mental health and drug and alcohol history was not collected for this study. Lower education level and unemployment prior to injury have been described as a prognostic factor for reduced health status postinjury due to the patients’ inability to adapt with the stressors associated with traumatic injury and access to a complex healthcare system [12, 29, 44, 45]. Patients become at risk of functional limitation with activities of daily living, psychological decline, and chronic pain [45]. Halvachizadeh et al. described the long-term psychiatric outcomes 20 years after polytrauma and found over 50% of patients showed symptoms indicative of clinical depression and anxiety [46]. Injury severity was not a predictor factor of psychiatric sequelae but non-injury factors such as preinjury psychiatric treatment or additional psychiatric insults [46]. It is unknown whether these patients postinjury have a poor, good, or equal health status compared to patients who did participate in the study. Strategies are needed to address the lack of availability to non-responders and identify predictors or sources for non-adherence, whether it be monetary or service provision. Further studies are required to extrapolate these findings and compare non-responders to other geographical areas.

Our study had some limitations. First is that the response rate to long-term follow-up for this prospective study was 64% which is on par with what we found in the literature [6, 10, 33]. There was responder bias, with non-responders being significantly younger, more likely to have a low initial GCS, lower education, and unemployed compared to the responders. Age was the probable reason for loss to follow as these young healthy patients were more likely to have recovered and returned to daily activities, so less likely to respond. Younger age group, higher prevalence of mental health, or excellent postinjury recovery may be reasons participants fail to follow-up as they no longer want to go through the trouble of doing another assessment [10]. Our non-responder cohort demonstrated similarities consistent with the literature regarding demographics and low follow-up rate, which may influence the results of the functional outcome studies as an incomplete representation of the group. Conversely, responders were older and more likely to have retired hence more likely to be available to answer follow-up phone calls or complete questionnaires. Lost to follow-up patient data is not uncommon in the literature and is unlikely to be missing at random as people may not respond for multiple different reasons.

Second is the use of the generic SF36 tool to evaluate HRQOL in trauma populations. Hoffman et al. state that although the SF36 is the most widely used generic tool to evaluate trauma population HRQOL outcomes, it captures only a small proportion of non-trauma specific health outcomes [47], leading to a lack of understanding of the impact of injury on different populations and failure to identify modifiable predictors of long-term recovery. However, using a standardised measure like the SF36, we can quantify the impact of severe injury in a way that can be comparable to other chronic health diseases. This not only helps in conducting more comprehensive and comparable research but also highlights the societal burden of injuries and helps in the recognition of polytrauma as a disease in our society. Third is the structure and length of follow-up. This study only captures participants up to 12 months postinjury via telephone follow-up. Our trauma centre provides follow-up by many specialities until those medically required and cannot be done by the patients’ general practitioners. We believe that there is a value in separating clinical and research functional assessment related to follow-ups to avoid potential bias inflicted on the participants. Longer follow-ups and monitoring for the increased prevalence of chronic physical health are needed to potentially characterise the quality of trauma survival and functional outcomes over time.

This study demonstrated the complexity of injury recovery and the challenges in reporting modifiable predictors of recovery. Although improvements in MCS and PCS were evident overtime in this cohort, the proportion of patients still experiencing reduced HRQOL below the Australian general population at 12 months was greater than 70% (Table 4). This significant reduction in physical and psychological capacity of trauma patients at 12 months postinjury meets the definition of chronic disease status, which could be predicted only by non-modifiable factors. Injury severity, polytrauma, low GCS on admission, ICU admission, hospital LOS, and compensable status are non-modifiable predictors, leaving primary prevention the only intervention to improve these outcomes. A fundamentally different approach is required to capture non-responders and identify modifiable predictors of long-term polytrauma outcomes. Further research and consensus are required to identify functional recovery outcome tools to specifically target potentially modifiable factors and develop more effective strategies for preventing chronic disease and improving the health outcomes of the polytrauma patient.

**Table 4:** Descriptive summary of variables associated with physical and mental functioning outcomes over time

		PCS mean (SD)			MCS mean (SD)		
Variable	Level	Baseline	6 months	12 months	Baseline	6 months	12 months
Age category	60 + years	48.6 (12.2)	33.7 (11.2)	37.2 (11.5)	52.7 (12.3)	44.5 (16.9)	47.6 (14.2)
	< 60 years	54.4 (8.2)	38.0 (10.9)	41.4 (11.0)	50.8 (11.6)	43.4 (15.1)	44.8 (14.7)
Sex	Female	51.9 (9.9)	34.9 (10.5)	40.5 (11.8)	51.6 (11.6)	41.7 (15.6)	44.0 (14.6)
	Male	53.2 (9.7)	36.9 (11.4)	39.6 (11.2)	51.3 (11.9)	44.5 (15.7)	46.5 (14.5)
Education	University	53.8 (7.9)	38.6 (12.0)	40.2 (12.5)	51.7 (9.1)	41.5 (17.3)	44.1 (15.2)
	No university	52.7 (10.0)	36.0 (11.0)	39.8 (11.2)	51.3 (12.2)	44.2 (15.4)	46.2 (14.4)
Employment	Full/part-time	54.1 (9.1)	37.1 (11.3)	40.3 (10.8)	52.1 (11.4)	44.2 (15.2)	46.6 (13.6)
	Not working	50.7 (10.3)	35.4 (11.1)	39.0 (12.2)	50.3 (12.4)	43.2 (16.4)	44.6 (15.8)
CCI	0	55.0 (8.4)	39.9 (11.0)	43.3 (10.5)	50.8 (11.8)	45.2 (13.6)	46.4 (14.2)
	1	54.0 (6.2)	34.8 (10.1)	39.7 (11.3)	52.1 (10.7)	40.9 (16.5)	42.9 (14.1)
	2 +	48.4 (11.7)	33.3 (10.9)	36.3 (11.3)	52.0 (12.3)	43.3 (17.4)	46.4 (15.1)
ISS category	> 15	53.1 (9.4)	36.6 (10.7)	39.5 (11.4)	51.3 (12.0)	42.8 (15.2)	45.3 (14.6)
	< 15	51.0 (11.6)	34.5 (15.4)	42.1 (11.2)	51.4 (10.6)	52.8 (18.0)	49.6 (14.0)
Initial GCS	≥ 13 (minor)	52.9 (9.5)	36.7 (11.5)	40.6 (11.5)	51.6 (11.6)	45.1 (15.4)	47.6 (13.6)
	9–12 (moderate)	51.8 (7.7)	34.1 (8.5)	34.0 (10.5)	49.3 (13.8)	33.8 (16.1)	42.1 (14.2)
	≤ 8 (severe)	52.5 (12.0)	35.3 (10.0)	36.3 (9.9)	50.9 (12.3)	39.1 (16.1)	35.9 (18.0)
Polytrauma	Yes	52.7 (9.5)	36.3 (10.8)	39.2 (11.3)	50.9 (12.1)	43.3 (15.7)	45.3 (15.0)
	No	53.3 (11.1)	36.8 (14.2)	43.7 (10.9)	53.8 (9.6)	47.2 (15.7)	49.5 (10.8)
Compensable	Yes	52.9 (8.9)	31.9 (9.2)	34.8 (9.0)	53.8 (8.6)	39.8 (16.7)	40.5 (15.9)
	No	52.8 (10.1)	38.6 (11.5)	42.4 (11.6)	50.2 (12.8)	45.7 (14.9)	48.6 (13.1)
Region of injury	Head/neck	53.4 (9.3)	36.2 (10.7)	38.9 (11.2)	50.8 (11.0)	41.6 (16.4)	43.8 (15.1)
	Face	55.6 (6.1)	36.8 (13.1)	39.8 (10.9)	55.6 (6.1)	36.8 (13.1)	39.8 (10.9)
	Chest	52.0 (10.2)	36.2 (11.1)	38.3 (11.4)	50.3 (12.5)	41.9 (15.8)	44.9 (15.1)
	Abdomen	52.4 (10.4)	36.5 (11.2)	39.1 (12.1)	50.9 (13.4)	42.9 (16.6)	46.4 (14.9)
	Extremities	52.9 (9.5)	35.3 (11.1)	39.2 (11.2)	51.1 (11.7)	44.1 (15.5)	46.0 (14.3)
	External	52.6 (9.4)	36.0 (10.5)	39.4 (11.6)	50.6 (12.0)	43.0 (15.3)	45.5 (13.7)
IRSAD quartile	4 (high)	57.2 (4.7)	38.2 (8.9)	48.0 (16.1)	51.3 (8.8)	44.1 (10.0)	47.8 (13.9)
	3	56.2 (4.6)	39.0 (12.4)	40.8 (12.8)	53.2 (7.4)	45.3 (11.8)	47.6 (8.9)
	2	51.6 (11.1)	35.9 (11.2)	38.8 (11.3)	50.5 (13.5)	43.8 (16.7)	45.9 (15.9)
	1 (low)	53.2 (8.6)	35.9 (10.7)	40.9 (10.4)	52.4 (9.9)	42.9 (16.1)	44.9 (14.1)
ASGS	Major cities	53.3 (8.7)	36.2 (11.8)	39.3 (11.9)	51.4 (10.5)	43.2 (15.2)	45.6 (14.4)
	Inner Regional	52.7 (9.9)	37.1 (10.9)	40.1 (10.9)	51.7 (12.8)	44.9 (15.6)	46.8 (14.2)
	Outer Regional	49.9 (14.1)	32.8 (8.0)	42.1 (10.7)	49.8 (14.6)	40.6 (15.7)	41.5 (17.8)

Descriptive summary for subgroups defined by variables associated with physical and mental functioning outcomes over time

## Supplementary Information

Below is the link to the electronic supplementary material.

Supplementary file1 (DOCX 32 KB)
